# The Role of IL-6 in Ischemic Stroke

**DOI:** 10.3390/biom15040470

**Published:** 2025-03-23

**Authors:** Hanna Pawluk, Alina Woźniak, Agnieszka Tafelska-Kaczmarek, Agnieszka Kosinska, Mateusz Pawluk, Krzysztof Sergot, Renata Grochowalska, Renata Kołodziejska

**Affiliations:** 1 Department of Medical Biology and Biochemistry, Faculty of Medicine, Collegium Medicum in Bydgoszcz, Nicolaus Copernicus University in Toruń, Karłowicza 24, 85-092 Bydgoszcz, Poland; pawluk.mateusz23@gmail.com (M.P.); renatakol@poczta.fm (R.K.); 2 Department of Organic Chemistry, Faculty of Chemistry, Nicolaus Copernicus University, Gagarina 7, 87-100 Toruń, Poland; tafel@umk.pl; 3 Centre for Languages & International Education, University College London, 26 Bedford Way, London WC1H 0AP, UK; a.kosinska@ucl.ac.uk; 4 Laboratory of Laser Molecular Spectroscopy, Institute of Applied Radiation Chemistry, Faculty of Chemistry, Lodz University of Technology, Wroblewskiego 15, 93-590 Lodz, Poland; krzysztofsergot@gmail.com; 5 Laboratory of Cell Biochemistry and Biology, Department of Biotechnology, Institute of Biological Sciences, Faculty of Biological Sciences, University of Zielona Góra, Prof. Szafran 1, 65-516 Zielona Góra, Poland; r.grochowalska@wnb.uz.zgora.pl

**Keywords:** ischemic stroke, interleukin 6, biomarker

## Abstract

The pathophysiology of a stroke is a complex process involving oxidative stress and inflammation. As a result of the actions of reactive oxygen species (ROS), not only does vascular damage occur, but the brain tissue is also damaged. It is a dynamic process, induced by a cellular–molecular immune response, focused on the development of an immediate reaction. During ischemia, inflammatory mediators are released, among which IL-6 plays a particularly important role in the acute phase of a stroke. Recently, a lot of attention has been devoted to this pleiotropic pro-inflammatory cytokine, which enhances the migration of leukocytes and is controlled by chemokines and the expression of adhesion handlers. The impact of IL-6 on the severity of neurological treatment and on patient prognosis in AIS is of interest to many researchers. More and more data indicate that it may be a reliable prognostic factor in strokes.

## 1. Introduction

A stroke is one of the most common diseases in the world, affecting people over the age of 40 and being a leading cause of disability and even death. According to data from the World Health Organization, one in six people worldwide will experience a stroke during their lifetime. The ischemic stroke is a type of stroke that affects about 85–90% of patients, and it can be caused by cerebral microangiopathy, arteriosclerosis, embolism, or blood clotting disorders [1,2].

So far, it has been proven that ongoing inflammation and oxidative stress play a significant role in the etiology of a stroke [3]. As a result of the obstruction of the cerebral artery, there is a lack of oxygen, glucose, and lipids, ultimately leading to energy failure. ATP depletion disrupts ion gradients, causing cellular depolarization, calcium influx, and excessive glutamate release. The activation of the *N*-methyl-D-aspartate (NMDA) receptors leads to further calcium influx and the expression of destructive enzymes [3].

Oxidative stress is one of the causes of the pathogenesis of ischemic neuronal damage, in which calcium levels mediate the process. Disruptions in calcium homeostasis and its increase in cells enhance the production of reactive oxygen species (ROS). These can also be generated by damaged mitochondria or through the activation of cellular receptors that transmit signals from inflammatory mediators [3]. In patients with acute ischemic stroke (AIS), ROS generated during the ischemia and reperfusion phases lead to brain damage. When the redox potential balance in the cell is disturbed, it causes the peroxidation of polyunsaturated fatty acids and leads to other intracellular damages, ultimately resulting in apoptosis [4,5,6].

Other mechanisms also contribute to brain damage following a stroke, such as neuroinflammation and disturbances in hormonal balance [7,8]. The inflammatory response, both at the periphery and within the central nervous system (CNS), involves various types of cells and molecular pathways, in which cytokines play key roles [8].

Although cytokines play an important role in the pathophysiology of stroke, the loss of balance between pro-inflammatory and anti-inflammatory cytokines after a stroke affects not only the extent of brain damage but also the recovery process. Importantly, cytokines, as pleiotropic biomolecules, participate in both innate and adaptive immune responses. As the stroke injury progresses, various signaling pathways are activated, modified, or interlinked. Pathways associated with the S1P receptor, particularly S1P1 and S1P3, are involved in modulating various aspects of neuroinflammatory responses. They participate in immune cell trafficking, blood–brain barrier (BBB) integrity, and cytokine production [8].

An important role in the innate immune response is played by the intracellular multi-protein complex, the NLRP3 inflammasome, which detects cellular damage and mediates the production of pro-inflammatory factors. The activation of NLRP3 occurs in stages, with the second stage involving the active inflammasome converting pro-caspase-1 into its active form, which triggers the pro-inflammatory cellular response by cleaving the precursors of interleukin-1 beta (IL-1β) and interleukin-18 (IL-18) into their mature forms [9,10,11].

Other signaling cascades include the mitogen-activated protein kinase (MAPK), Janus kinase (JAK)/signal transducer and activator of transcription proteins (STAT3), transforming growth factor β (TGF-β)/Smad protein, high mobility group box 1 protein (HMGB1)/toll-like receptors (TLR), and nuclear factor kappa-light-chain-enhancer of activated B cells (NF-κB). All changes in the expression, organization, activation, or stimulation of these signaling pathways can occur at any stage of the developing inflammatory condition. There may be an increased secretion of cytokines that promote the inflammatory response and influence the course and progression of a stroke [8].

The released cytokines are recognized by specific membrane receptors, which transmit signals to the cell nucleus, where there is an upregulation of genes encoding acute phase proteins (APP). This particularly applies to pro-inflammatory cytokines responsible for inducing, mainly in the liver, the fibrinogen and c-reactive protein (CRP), a standard inflammation marker associated with stroke [1]. It has been shown that the increase in the acute phase immune response factor, stimulated by interleukin 6 (IL-6), can rise not only during the acute phase of stroke but also in response to a broad spectrum of systemic inflammatory conditions [12].

Many studies suggest that anti-inflammatory action can significantly reduce cardiovascular risk. However, there is limited information, and when available, the findings are inconclusive regarding the ongoing inflammatory process in stroke involving this cytokine. Data from several human studies suggest a potentially causal role of IL-6, a pro-inflammatory cytokine, in vascular disease [13,14], which may make it a promising therapeutic target.

In addition, the use of an anti-inflammatory treatment targeting IL-6 may seem like an important tool in the treatment of strokes. Cohort studies have managed to show strong associations between circulating IL-6 levels and the risk of coronary artery disease [15], but there is limited evidence regarding its associations with ischemic stroke [16,17].

There are also reports suggesting that it could be a reliable prognostic factor in the acute phase of AIS and a biomarker in the development of clinical complications resulting from acute ischemia [2]. We decided to use pooled data from the published literature through a systematic review and meta-analysis to investigate the relationship between circulating IL-6 levels and the risk of ischemic stroke in humans. Additionally, we aimed to examine the correlation between serum IL-6 levels, the severity of neurological deficits, and patient prognosis after AIS, depending on stroke volume.

## 2. The Role and Properties of Cytokine IL-6

IL-6 is a cytokine involved not only in inflammatory and infectious responses but also in the regulation of metabolic, regenerative, and neuronal processes. The regenerative or anti-inflammatory effects of this cytokine are associated with classical signaling, while trans signaling is more likely involved in pro-inflammatory processes of IL-6 [18].

IL-6 can be secreted by both immune cells (T lymphocytes, B lymphocytes, macrophages, and microglia) and non-immune cells (muscle cells, adipocytes, fibroblasts, endothelial cells, and neurons) [19,20,21].

Microglia and astrocytes produce IL-6 in response to brain injury, which may contribute to both chronic inflammation and regeneration, clearance of debris, or tissue repair [22,23]. Astrocytes secreting IL-6 promote the polarization of Th1 to Th2, mediate an immunosuppressive microenvironment, and contribute to neurogenesis, angiogenesis, and neuronal differentiation [24].

Endothelial cells secreting IL-6 increase vascular permeability and promote the early transcriptional activation of genes related to angiogenesis through stimulation of STAT3 phosphorylation [25]. At the same time, the IL-6 receptor (IL-6R) activates angiogenesis-related pathways such as phosphoinositide 3-kinase (PI3K)/serine/threonine kinase (AKT) and JAK/STAT [26]. By increasing angiogenesis, IL-6 enables neuronal regeneration and recovery of function during a stroke. It also facilitates post-traumatic healing in the CNS by repairing endothelial cells and promoting revascularization of brain blood flow in the period after ischemic stroke. However, excessive activation of angiogenesis can lead to brain edema [27].

IL-6 may support neuronal survival after ischemic injury [28]. Secreted by immune system cells (macrophages, T lymphocytes), it activates the inflammatory response and intensifies stroke-related changes, which may exacerbate brain damage [29].

The IL-6 molecular weight ranges from 20 to 30 kDa, depending on the cellular source. It is a type of multi-effector cytokine and, as a pleiotropic cytokine, plays an important role in host defenses [30].

IL-6 affects immune responses and regulates hematopoiesis in an autocrine or paracrine manner. It plays an important role in the growth, differentiation, regeneration, and degradation of nerve cells in both the peripheral and CNS.

IL-6 is not only responsible for the activation and stimulation of neutrophils and monocytes but also enhances endothelial cells to secrete adhesive molecules and inflammatory mediators and strengthens the local inflammatory response [31].

It has also been found that IL-6 is an important factor in the differentiation and growth of hematopoietic cells as well as B and T cells, endothelial cells, and osteoclasts. It can induce the production of the fibroblast growth factor, stimulate macrophage colony formation, promote tumor necrosis factor (TNF) production, and participate in the proliferation of smooth muscle cells [32]. On the other hand, TNF-α can stimulate brain pericytes to synthesize IL-6 through the activation of NF-κB. The soluble form (sTNF-α) acts at the systemic level, enhancing the action of macrophages and the expression of cytokines IL-6 and IL-1 [33]. Additionally, IL-6 facilitates the post-traumatic healing of CNS and endothelial cell repair [27,34]. This cytokine increases the survival of neurons and reduces their excitotoxic damage caused by injury or protects neurons from apoptosis [35].

It has been shown that the high-sensitivity C-reactive protein, IL-6, IL-1 receptor antagonist (IL-1Ra) [36,37], lipoprotein-associated phospholipase A2 (Lp-PLA2-A) [15], and chitinase-3-like protein 1 (YKL-40) [38,39] are associated not only with the initial but also with subsequent strokes. IL-6 is involved in the etiology of atherosclerosis and the stability of atherosclerotic plaques, which may influence the development of a subsequent stroke [40,41].

Furthermore, atherosclerosis of large arteries and the obstruction of small vessels share several common factors that may contribute to the development of atherosclerosis, which in turn is a cause of certain subtypes of strokes [42,43].

It has been demonstrated that the correlations between IL-6 and YKL-40 are more pronounced in patients with large artery atherosclerosis and small vessel occlusion. These markers are associated with recurrent stroke, complex vascular events, and poor functional outcomes [44,45].

## 3. IL-6 as a Mediator of Inflammatory Response in Stroke

In ischemic stroke, inflammation and oxidative stress play a key role in neuronal cell apoptosis. IL-6 is crucial in this complex, multi-stage pathophysiological process due to its synthesis in CNS and its ability to modulate inflammation through dual signaling pathways [46,47,48]. Depending on the presence of IL-6R or the membrane-associated signal transducer gp130, IL-6 signaling can lead to pro-inflammatory and anti-inflammatory responses [48].

IL-6 utilizes two unique signaling pathways: classical and trans signaling. In the classical pathway, IL-6 binds to IL-6R on the cell surface, forming a hexameric complex with two IL-6 molecules, two IL-6Rα subunits, and two gp130 subunits. This pathway primarily occurs in specific immune and non-immune cells such as T lymphocytes, hepatocytes, and neutrophils, and has an anti-inflammatory effect [49,50].

In contrast, IL-6 also activates pro-inflammatory trans signaling where it binds to the soluble isoform of IL-6R (sIL-6R), which lacks the transmembrane domain. This complex, IL-6: sIL-6R, can bind and activate gp130, triggering a broader cellular response. Natural inhibitors like soluble gp130 (sgp130) limit this trans signaling [49,51].

Unlike classical signaling, trans signaling does not require membrane-bound IL-6Rα for the indicator initiation, expanding the range of target cells to almost all cells in the body [52]. The soluble IL-6:IL-6Rα complex can initiate IL-6 signaling in cells containing the signal transducer gp130, which is present in neurons, neuronal cells, endothelial cells, and oligodendrocytes [52]. Both mechanisms contribute to neuroinflammation after stroke, but trans signaling is considered the dominant pathway in the brain [49,53].

While trans signaling aids in damage repair after stroke, it can also trigger harmful recurring inflammatory responses. It is linked to ischemic stroke risk due to its strong pro-inflammatory and pro-atherogenic effects [54].

**Figure 1:**
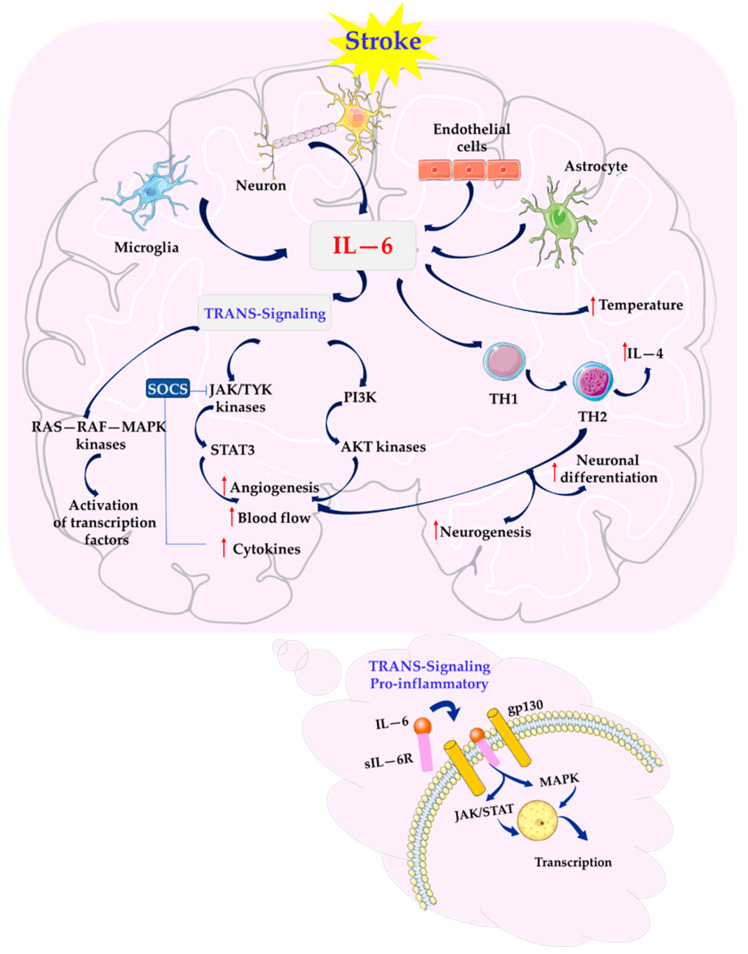
IL-6 trans signaling. The IL-6/sIL-6R/gp130 complex can activate intracellular tyrosine kinases that activate STAT transcription factors or the RAS-RAF-MAPK or PI3K pathway. Trans signaling can lead to the expression of a pro-inflammatory state via increased JAK/STAT signaling due to SOCS inhibition. STAT3 phosphorylation can enhance the transcription of genes related to angiogenesis and intense cerebral blood flow. AKT—protein kinase B; IL-4—interleukin 4; IL-6—interleukin 6; JAK—Janus kinase; pg130—signal transducing receptor; PI3K—phosphatidylinositol 3-kinase; RAS—RAF—MAPK—small guanosine triphosphatases—serine/threonine-specific protein—mitogen-activated protein kinases; sIL-6R—soluble isoform of IL-6 receptor; SOCS—proteins called suppressors of cytokine signaling; STAT3—signal transducer and activator of transcription 3; TH1, TH2—lymphocytes; TYK—tyrosine kinase. This figure was created using Servier Medical Art (available at https://smart.servier.com/) (accessed on 12 September 2024).

Both signaling pathways activate intracellular kinases, including JAK (and to a lesser degree, TYK), which further activate proteins like the transcription factors STAT, the RAS-RAF-MAPK pathway, and PI3K (Figure 1) [30,32,55].

Trans signaling notably boosts JAK/STAT signaling, surpassing MAPK, by inhibiting suppressors of cytokine signaling (SOCS) and affecting NFκB. SOCS3 is a negative regulator of STAT3, which affects the reduction in IL-6 signal transduction and interferes with immune cell migration [49,56].

The increased activity of JAK/STAT caused by the lack of feedback regulation promotes cytokine expression through chronic activation of microglia and astrocytes. Excessive microglial activation leads to a phenotypic shift to pro-inflammatory M1, which is manifested by the increased expression of inflammatory markers and pro-inflammatory cytokines, triggering nerve inflammation [56].

Astrocytes can release pro-inflammatory cytokines and chemokines that recruit microglia, collectively contributing to the damage of BBB [56,57].

Disruption of BBB integrity allows the infiltration of peripheral immune cells, which release pro-inflammatory mediators. Persistent nerve inflammation causes oxidative stress, synaptic dysfunction, and neuronal death [56,58].

IL-6R also plays a role in angiogenesis after stroke by activating the JAK/STAT and PI3K/AKT pathways. IL-6 promotes STAT3 phosphorylation and the transcription of angiogenesis-related genes, enhancing neovascularization and cerebral blood flow in the post-ischemic period [26,30,59].

Astrocyte-derived IL-6 further contributes to angiogenesis, neurogenesis, and neuronal differentiation by promoting the polarization of TH1 lymphocytes into TH2, facilitating recovery after stroke [24,60,61,62]. Astrocytes, through IL-6 regulation, support neurovascular regeneration and functional recovery post-stroke [24].

## 4. IL-6 and Cognitive Functions in Stroke

Almost 1/3 of stroke patients develop cognitive impairments. Post-stroke cognitive impairment (PSCI) is a common complication after a stroke and directly affects functioning and quality of life, including the ability to work and maintain interpersonal relationships. In patients with PSCI, there is a decline in short- and long-term memory, reasoning, coordination of movements, and task planning. Around 20–30% of these patients develop dementia [63,64]. Post-stroke cognitive impairments contribute to the growing health, social, and economic burden [65].

It is not always easy to diagnose and assess the degree of PSCI based on clinical assessment, imaging, and neuropsychological tests, which is why inflammatory mediators and brain damage markers can help diagnose and predict cognitive function impairment [63,64]. It has been shown that memory deficits after acute brain ischemia are associated with increased levels of pro-inflammatory cytokines (IL-1, IL-6 and TNF-α) in the brain and neurodegeneration [65,66].

It is known that IL-1, IL-8, TNF-α, and IL-6 can be predictors of cognitive decline in late middle age [67,68,69].

Higher levels of IL-6 in serum are associated with a greater decrease in psychomotor speed [70]. It seems that elevated IL-6 levels may cause microvascular changes, leading to reduced neuronal propagation and impaired processing speed [67]. IL-6 can directly activate endothelial cells, which secrete several types of cytokines and chemokines, initiating a cytokine storm. A complication of the cytokine storm is the dysregulation of endothelial cells, characterized by abnormal coagulation and vascular leakage [71].

Furthermore, the dysregulation of IL-6 along with other pro-inflammatory cytokines, such as IL-1 and TNF-α, can lead to nerve inflammation, which is associated with neurodegenerative diseases such as Alzheimer’s disease (AD), Parkinson’s disease (PD), depression, and multiple sclerosis (MS) [72].

IL-6 stimulates the synthesis of the beta-amyloid precursor protein (Aβ) and exacerbates tau pathology in AD, enhancing neuronal damage. It contributes to cognitive decline by disrupting the integrity of BBB, impairing neuronal survival, and synaptic plasticity [28,49,70].

However, not all studies confirm the link between IL-6 levels and cognitive decline [73].

The risk of cognitive impairment with a stroke increases 5 to 8 times [65,66]. The cause of this is inflammation, which plays a role in the pathogenesis of post-stroke cognitive impairment. IL-6, along with other inflammatory cytokines such as TNF-α and IL-1β, may play an important role in cognitive impairments following a stroke, as they are key regulators of the acute phase response to inflammation and tissue damage. The levels of pro-inflammatory cytokines, including IL-6, are elevated in patients with PSCI [65,66,74,75,76,77,78,79,80].

Therefore, IL-6 may be responsible for cognitive disturbances after a stroke, as it is one of the main regulators of the acute-phase response to inflammation and tissue damage, and its concentration is elevated in patients with PSCI [44,45,49,50,51,52,53,54,55].

During ischemia, IL-6 activates microglia, autophagy, and endoplasmic reticulum (ER) stress, which leads to hippocampal neuron death and cognitive decline [81].

IL-6 is also one of the factors influencing demyelination and myelin regeneration following damage to the white matter integrity after a stroke. An increase in IL-6 levels in neurons at risk of ischemia may be associated with neuroprotection; however, excessive production of IL-6 in the brain leads to neurodegenerative changes [82].

It has been found that elevated IL-6 levels released by activated microglia in the central nervous system are associated with early neurological damage [83]. High concentrations of IL-6 may therefore be an important factor promoting the development of cognitive impairments and may be linked to their prevalence [79]. According to the Montreal Cognitive Assessment (MoCA), increased IL-6 levels after 18 months were associated with cognitive dysfunction in a 36-month follow-up of patients hospitalized for acute stroke [75]. Similarly, an increase in interleukin-6 levels was associated with a decline in cognitive functions according to MoCA, assessed between 1 year and 3 months, in patients with AIS or transient ischemic attack (TIA) [76]. The risk of cognitive deterioration after stroke increased by as much as 95% for the highest IL-6 levels compared to the lowest [76].

Elevated IL-6 levels may also be associated with psychological complications, the occurrence of anxiety or depression, which negatively affect rehabilitation outcomes, the effectiveness of neurotrophic agents, and even mortality in patients with AIS [78,79]. IL-6, along with IL-1β, TNF-α, and interleukin-17A (IL-17A), can regulate the enzyme 2,3-indoleamine-2,3-dioxygenase, thereby modulating serotonin, which is associated with the pathogenesis of psychological complications [78]. It has also been observed that with increasing IL-6 levels in plasma, the risk of post-stroke delirium rises, which is characterized by acute, fluctuating changes in attention, awareness, and cognition. Hence, both immunodepression and systemic inflammation, reflected by increased IL-6 levels, are associated with poor cognitive outcomes [77]. Chronic inflammation, in turn, can activate neurotoxic pathways, and damaged neurons may exacerbate neurological disturbances by producing chemokines, activating microglia, and astrocytes [84].

In general, pro-inflammatory cytokines, including IL-6, cross the blood–brain barrier, promoting communication between the central nervous system and the periphery, and causing brain damage. Additionally, hyperperfusion caused by a stroke leads to endothelial damage, which promotes nerve inflammation, disrupts the blood–brain barrier, and causes neurodegeneration [67,85].

However, the relationship between IL-6 and cognitive decline after a stroke is not fully established [86,87]. In a study examining the relationship between inflammatory markers and baseline cognitive status, as well as subsequent cognitive decline, it was found that interleukins IL-8 and IL-12, rather than IL-6, are independent predictors of cognitive deterioration [83]. Other studies have found that the levels of Th17-related cytokines, including IL-6, in serum, had minimal predictive value for the recovery of cognitive functions during subacute hospital rehabilitation after a stroke [88]. Central nervous system inflammation may likely mask the relationship between systemic inflammation and cognitive functions [83].

Despite the ambiguous clinical studies, it seems reasonable to assess the levels of inflammatory biomarkers, including IL-6, in patients with PSCI. These studies, combined with neuroimaging and neuropsychological evaluation, may help in the early detection of PSCI and in preventive actions against post-stroke cognitive impairments [65]. The measurement of circulating IL-6 could be used as a screening test to identify AIS patients with cognitive impairment [89]. This test can be extended to assess other pro-inflammatory cytokines, such as TNF-α, IL-8, and IL-17A, which positively correlate with anxiety, depression, or cognitive impairment at various time points after the onset of AIS [71,75].

## 5. Circulating IL-6 and the Risk in AIS

Interleukin-6 plays an important role in modulating the inflammatory response of the nervous system in stroke. It influences the promotion of homeostasis in response to immune system stimulation through the classical path. The homeostatic, anti-inflammatory route affects nerve regeneration and tissue repair [46,53]. On the other hand, the trans pathway, due to the activation of pg130 on neuroinflammatory cells, can intensify inflammation and worsen stroke outcomes [46].

IL-6 plays a role in the acute phase of stroke as a mediator of the inflammatory process, a reliable prognostic factor, and a neurotrophic factor in the later stages of brain ischemia development [35,90].

IL-6 is a key marker of inflammation, and its concentration rises dynamically in serum within a few hours of AIS and can remain elevated for up to 3 months after the stroke [2,16,91,92,93,94,95,96,97,98,99,100,101,102,103,104,105,106,107].

In population studies in humans, it has been shown that higher levels of IL-6 are associated with stroke risk factors and with a higher risk of AIS occurrence [12,16,30,45,108,109,110,111,112,113,114,115,116,117,118]. IL-6 levels can be useful as an analytical biomarker for stroke recurrence after AIS [108], and its concentration reflects ethnic differences [16,119]. Jiang et al. found that the risk of stroke recurrence rises by 8% for every 1 pg/mL increase in serum IL-6 levels, both in unadjusted and adjusted analyses [108]. A meta-analysis conducted by McCabe et al. demonstrated that inflammatory biomarkers’ levels, including IL-6, were independently associated with vascular recurrence after stroke. For every threefold increase in IL-6 levels, there was a 20% to 25% increase in the risk of major cardiovascular events [110]. Multivessel disease (PolyVD) combined with elevated IL-6 levels (≥2.64 pg/mL) facilitates the risk of stroke recurrence in patients with AIS or TIA after one year of observation [120]. Additionally, IL-6 promoter polymorphisms predict recurrent young strokes. During one year of observation of patients with different IL-6 promoter genotypes, it was found that the frequency of recurrent strokes in young individuals with moderate internal carotid artery stenosis increased for the IL-6 G allele promoter [109].

Elevated levels of IL-6 are associated with unsuccessful reperfusion in patients with AIS and large vessel occlusion (LVO) treated with mechanical thrombectomy (MT) [121]. On the other hand, decreased IL-6 levels at admission were associated with the first-pass effect (FPE), meaning complete or nearly complete reperfusion achieved after a single thrombectomy. IL-6 thus enables the differentiation between FPE and non-FPE, with the optimal threshold for its level being 3.0 pg/mL [122].

Interleukin-6 may have a major impact on the prognosis in AIS [2,91,92,93,95,96,99,103,115,120,123,124,125,126,127,128,129]. Elevated levels of this marker are correlated with the severity of neurological damage both clinically (National Institute of Health Stroke Scale (NIHSS); Scandinavian Stroke Scale (SSS); European Stroke Scale (ESS); Canadian Neurological Scale (CNS)) and radiologically, with diagnostic subtypes (according to the Trial of Org 10172 in Acute Stroke Treatment (TOAST) classification or Oxfordshire Community Stroke Project Classification (OCSP)), activation of the acute phase immune response, and poor prognosis [2,91,92,93,95,96,99,103,104,105,115,116,120,123,124,125,126,127,128,129].

It has been shown that an increase in IL-6 levels in the serum within the first 24 h after a stroke is associated with worsened functional status and poorer neurological outcomes in patients [2,91,95,130], as well as with the volume of damage [131,132,133].

A similar correlation has been established for IL-6 in the cerebrospinal fluid of stroke patients. Increased IL-6 levels in the cerebrospinal fluid at 6 h post-stroke have been shown to correlate with the degree of neurological deficit, assessed on days 1 and 7 post-stroke using the NIHSS, as well as with functional deficits measured by the Barthel index (BI) [134].

Recent studies also suggest that elevated levels of IL-6 and TNF-α assessed on days 1 and 7 significantly correlate with greater stroke severity and worse functional outcomes, as indicated by higher NIHSS and mRS scores. These findings align with studies indicating strong associations between inflammatory cytokines and post-stroke disability, further emphasizing their importance as prognostic indicators [2,135].

Higher IL-6 levels are associated with in-hospital neurological deterioration (ND) [103,136]. IL-6 may be an independent prognostic factor for ND in patients with AIS with large artery atherosclerosis (LAA) and small vessel occlusion (SVO) [103]. IL-6 levels may be independently associated with infarct volume, indicating brain infarction extension without the need for neuroimaging techniques [124]. It can help diagnose ischemic stroke and predict its severity by increasing the sensitivity/specificity of another non-invasive biomarker, miR-221 [123]. Elevated IL-6 levels after a stroke may be associated with mortality, adverse short- and long-term outcomes, and functional status, assessed using the modified Rankin Scale (mRS) at 3 months and 1 year [2,45,91,96,103,106,107,114,116,123,124,125,126,137]. An increase in IL-6 concentration was associated with an increased risk of stroke recurrence and disability within 90 days, with stroke recurrence mediating less than 20% of the association between IL-6 and functional outcome [114]. Therefore, the level of this biomarker can be an independent predictor of poor post-stroke outcomes [107,116,120,125] and is associated with a lower likelihood of a favorable outcome (mRS 0–2) [127]. It may also be a good candidate as a biomarker for individuals at risk of stroke-associated infection (SAI), enabling early identification of those at risk of infection, which consequently reduces the risk of premature death and hospitalization, especially in older adults [138]. A relationship was also shown between selected polymorphisms in IL-6 genes and outcomes after AIS and adverse effects (ADE). Individuals with the CC or GG genotype of the IL-6-174 G/C polymorphism had significantly lower NIHSS scores and poorer prognosis [139].

IL-6 plays a role in the pathogenesis of stroke, especially the atherosclerotic stroke of large arteries, and is associated with disease progression and functional outcomes within 3 months of the stroke [44]. There is also a relationship between the functional genetic variant of the interleukin 6 receptor and the risk and outcome of LAA stroke [140]. A causal relationship has been found between IL-6 levels and the severity of stenosis, susceptibility to atherosclerotic plaques, and long-term progression of the atherosclerotic plaque in the carotid artery [31,141].

Along with the increased IL-6 concentration, there is an increase in other inflammatory biomarkers, including C-reactive protein, fibrinogen, IL-1 receptor antagonist, and TNF-α. IL-6 can induce further phases of inflammatory markers and influence the prognosis of patients after AIS [2,35]. Low production of pro-inflammatory cytokines may be associated with relatively good outcomes if immunodeficiency is not accompanied by systemic inflammation linked to high circulating IL-6 levels. A multi-marker assessment based on cytokines may, therefore, be significant in predicting stroke outcomes by enhancing the prognostic value of the clinical model [142].

However, some reports do not confirm such correlations. According to Bustamante et al., based on a meta-analysis of 24 studies, it was found that IL-6 levels are associated with poor outcomes after stroke, but its predictive value was moderate, suggesting that IL-6′s practical application in clinical practice is unlikely [130]. It was also found that increased IL-6 expression indicates an inverse correlation with stroke severity, infarct size [143], short-term prognosis [144], and post-hospital improvement, assessed between discharge and 3 months [136]. Cytokines can contribute to both neurotoxicity and neuroprotection, locally as well as systemically, which is related to the ischemic damage timeline [46,118,143].

The level of IL-6 is directly associated with both fever and the strength of the acute phase response, so excluding patients with fever or infectious diseases may explain the protective effect of IL-6 on specific ethnic populations [118].

In summary, in Table 1, the concentrations of IL-6 and its impact on the risk of ischemic stroke, the degree of neurological damage, and functional outcomes after AIS are presented.

## 6. Modulation of IL-6

IL-6 represents a complex therapeutic target, as it plays both a toxic, inflammatory role and a regenerative role in the pathophysiology of stroke. Preclinical studies related to the inhibition or amplification of IL-6 are ambiguous [37,38,39,40,41,42]. It has been shown that IL-6 is a pro-inflammatory interleukin, which is why it is believed that reducing its expression is beneficial for neurological recovery. However, some of the literature reports suggest neurological benefits from upregulating this cytokine [32]. A detailed study of IL-6, focusing on the timing of its administration and the dosing regimen after stroke, may reveal the optimal strategy for achieving therapeutic effects. Many researchers have shown that manipulating its levels can impact the prognosis of patients [34,35,36] and improve post-stroke recovery care [13,30].

Controlling its concentration at different time intervals could be significant in post-stroke therapies. Experimental and preclinical studies have shown that inflammatory therapies, which reduce the number of serious adverse cardiovascular events (MACE) in coronary artery disease or stroke, can be used in stroke treatment. Establishing a specific relationship between inflammatory markers and the risk of recurrence of ischemic events may be useful in stroke-related anti-inflammatory therapies [145].

This “inflammatory hypothesis” was the basis for several clinical studies using different drugs in patients with coronary artery disease, and colchicine has shown promising therapeutic potential so far. In the CONVINCE study (Colchicine for Prevention of Vascular Inflammation in non-cardio Embolic Stroke) evaluating the impact of colchicine on stroke recurrence, its role in reducing both the inflammation caused by atherosclerosis and in stabilizing atherosclerotic plaques was demonstrated. Colchicine inhibits the function of neutrophils and reduces their ability to migrate to the site of inflammation, thus shortening the duration of the inflammatory response. Reduction in the production of inflammatory cytokines such as IL-1, IL-6, and TNF-α is also a result of the anti-inflammatory effects of colchicine. Therefore, daily use of colchicine may provide benefits in preventing recurrent strokes and major vascular events compared to usual care [45,146].

In another large-scale study, CANTOS (Canakinumab Anti-inflammatory Thrombosis Outcome Study), it was observed that the monoclonal antibody (canakinumab) had a beneficial effect on the final cardiovascular outcome, including stroke, in patients who had an unsuccessful myocardial infarction [147].

However, the monoclonal antibody was not able to show effectiveness in every stroke due to limited power and non-atherosclerotic causes of stroke. These observations suggest that monoclonal antibodies targeting IL-6 may be a promising tool in preventing ischemic stroke in patients with carotid artery atherosclerosis. The threshold of 2.0 pg/mL may help identify individuals who would benefit from anti-IL-6 drugs to prevent stroke [19,31].

So far, it has been shown that in stroke patients, higher levels of IL-6 in plasma correlate with increased stroke severity and poor long-term prognosis. Treatment with an immunosuppressive drug approved by the Food and Drug Administration (FDA) could block IL-6 receptors to inhibit its pro-inflammatory action. In an experimental study, researchers demonstrated that following the use of an immunosuppressant, young male mice with ischemic stroke showed a reduction in the infarct size. This led to the conclusion that controlling cytokine signaling, influenced by temporal and environmental changes, is crucial for improving post-stroke patient outcomes [148].

Depending on the timeline of ischemia, a direct injection of IL-6 into the brain after ischemia can reduce ischemic brain damage [35]. IL-6 has been found to play an anti-inflammatory role in both local and systemic acute inflammatory responses by regulating the levels of pro-inflammatory but not anti-inflammatory cytokines. This is supported by other literature reports, indicating that IL-6 is a promising tool in reducing the risk of stroke [12,35,45,149].

Endogenous IL-6 may play a key role in preventing neuronal damage during the acute phase of brain ischemia by activating the signaling protein and STAT3. The administration of a monoclonal antibody against the mouse IL-6 receptor (IL-6RA) 6 h after MCAO resulted in a significant reduction in the amount of phosphorylated STAT3 in the peri-infarct area of the cerebral cortex. After 24 h from MCAO, blocking IL-6 signaling led to an increase in the number of apoptotic cells in the peri-infarct area and an enlargement of the infarct size, as well as adversely affected neurological functions [150].

Another stroke therapy utilizing the potential of IL-6 could be modulation of the trans signaling pathway, enhancing trans signaling at an early stage or suppressing it at a later stage, to avoid a neuroinflammatory response [34,53,54].

It is known that in trans signaling, sIL6R, by forming a binary complex with IL-6, can activate gp130, triggering a pro-inflammatory cascade. However, if IL-6: sIL6R forms a ternary complex with soluble sgp130, the pro-inflammatory cascade is inhibited. The formation of the IL-6:sIL6R:sgp130 complex prevents the binding of gp130 and IL-6 trans signaling [22,53].

The ratio of the pro-inflammatory binary complex to the three-component complex (B/T) could be a new biomarker for assessing the increased risk of ischemic stroke, particularly of atherosclerotic origin, as the IL-6 trans signaling pathway is primarily associated with atherosclerotic cerebrovascular disease [54,151].

As a result of assessing the B/T ratio, it is possible to measure IL-6 signaling at different time points after ischemic injury. This could directly influence the achievement of the desired therapeutic effect by individualizing stroke therapy to support the body’s natural responses to reduce ongoing inflammation [152].

Knowledge of an elevated B/T ratio could enable clinicians to decide on the implementation of therapies using IL-6 trans signaling antagonists, such as spg130, which could lead to optimal modulation of the inflammatory signaling pathway [53].

Therefore, consideration could be given to introducing cell-based therapies into stroke treatment that can respond to local environments and increase or decrease IL-6 signaling. Stem cell-based therapies have a high therapeutic potential for regulating IL-6 signaling. This approach is currently receiving more attention in preclinical strategies, but such therapy with stem cells has not yet been implemented in stroke patient treatment [46].

Stem cells can be obtained at various stages of differentiation, and in the human body, they can be transformed into different cell types. They can show effective therapeutic results in the treatment of brain stroke or other diseases. After ischemic injury, they can be used to enhance any signaling pathway that would be most beneficial in treatment, considering the timeline of hypoxia [46]. It has been shown that treatment with embryonic stem cells and umbilical cord-derived stem cells (hESC-MSC-CM) leads to neurogenesis, angiogenesis, and protection of the brain tissue from ischemic damage. Inflammation and apoptosis are inhibited [152].

Additionally, pre-conditioning with IL-6 increases the efficacy of cell transplantation therapy in ischemic stroke by protecting neural stem cells (NSCs) from reperfusion injury. Conditioned NSCs promote angiogenesis, reduce infarct size, and improve behavioral outcomes [153].

Similarly, in an experimental study on mice with middle cerebral artery occlusion (dMCAO) receiving mesenchymal stem cell (HUMSC) transplants, which were protected by the caspase-1 inhibitor (VX765), their anti-apoptotic effect was demonstrated. This led to the activation of autophagy through the AMPK/mTOR signaling pathway both in vivo and in vitro [154].

Transplantation of human bone marrow-derived stem cells (BMSC) in ischemic stroke leads to an increase in T regulatory cells (Tregs), active mediators of immunomodulation, enabling neuroprotection, which is partly due to the anti-inflammatory properties of the cells in vitro. Regulatory T cells exert neuroprotective effects in stroke by inhibiting both inflammation and the activation of effector T lymphocytes [155].

Culturing oligodendrocyte precursor cells (OPC) with BMSC and Tregs show greater myelination potential and an increased production of IL-6 and basic fibroblast growth factor (FGF-β) compared to OPC cultured with BMSC alone. This indicates the potential of OPCs in an ischemic environment by modulating optimal IL-6 signaling pathways [46,156].

In further experimental animal studies, the injection of human neural stem cells into the hippocampus of mice reduced inflammation in ischemia–reperfusion brain injury by reducing pro-inflammatory cytokines and attenuating damage to BBB [157].

Similarly, the administration of adipose-derived mesenchymal stem cells (ADMSC) reduced neurological severity, limited the infarct area, and decreased cell apoptosis. They altered IL-6 signaling to restore BBB integrity and reduced the infiltration of peripheral inflammatory cells [158].

It has been demonstrated that the administration of NSC and NSC-1 cells 24 h after stroke led to improvements in motor and behavioral neurological functions, as well as a reduction in infarct size, which persisted for the following 7 days after the ischemic event. The authors concluded that the therapeutic effect was influenced by the increased levels of fibroblast growth factor 2 (bFGF) and IL-6 secreted by NSC-01. These results suggest that IL-6 and bFGF mediate the mechanism responsible for the improvement observed after in vitro MSC treatment [159].

## 7. Summary

In this study, we aimed to present the functional role of interleukin IL-6 and its association with ischemic stroke. Based on the available literature, it has been established that hypoxia can lead to various types of cellular damage and induce an immune response in which IL-6 amplifies the local inflammatory response. IL-6 activates the JAK/STAT pathway to promote the expression of pro-cytokines. The study attempted to demonstrate that IL-6 could serve as a reliable prognostic factor in the acute phase of AIS, as well as a biomarker for the development of clinical complications resulting from acute ischemia, or to assess the severity of neurological deficits and the prognosis of patients after AIS.

However, developing effective anti-inflammatory methods for preventing stroke remains an important, yet still unresolved clinical issue.

Understanding the temporal profile and cellular sources of IL-6, as well as its potential transport to the ischemic region in the early phase after a stroke, is crucial for comprehending how this cytokine interacts with potentially salvageable neurons in the ischemic penumbra. However, selecting the appropriate therapy requires consideration of the patient diversity in stroke cases. Monitoring IL-6 levels across various time points in different patients will enable more precise targeting of anti-IL-6 therapies, minimizing systemic side effects. Individual factors such as age, comorbidities, and genetic background can also affect the body’s response to IL-6 therapy and treatment. Moreover, inhibiting IL-6 signaling pathways must be approached cautiously. Rather than completely blocking IL-6, it may be more beneficial to focus on therapies that specifically target trans-signaling.

It is also important to consider the possibility of determining a specific relationship between this inflammatory marker and the risk of recurrence of ischemic events, so that it could be used in anti-inflammatory therapies for stroke patients. However, further research is needed to better understand which IL-6 signaling pathway is utilized at different time points after stroke to effectively support patient recovery.

## Figures and Tables

**Table 1:** The influence of IL-6 concentration on the risk of ischemic stroke, the degree of neurological damage, and functional outcomes after AIS.

Collection Time	Average Concentration	Control	Results	Conclusions	Reference
from 5 to 7 days	Peak plasma IL-6 30.5 pg/mL (*n* = 37)	–	Peak plasma IL-6 concentration above the median sample (30.5 pg/mL) at 5 to 7 days correlated significantly with cerebral infarct volume on computed tomography (r = 0.62, *p* = 0.006), mRS at 3 months (r = 0.62, *p* = 0.002), mRS at 12 months (r = 0.77, *p* < 0.001), stroke severity (r = 0.66, *p* < 0.001), Barthel index (BI) at 3 months (r = −0.74, *p* < 0.001), BI at 12 months (r = -0.77, *p* < 0.001) and was associated with increased mortality at 12 months (*p* = 0.0011).	Peak plasma IL-6 concentration in the first week of ischemic stroke significantly correlated with infarct volume, stroke severity, and clinical outcome at 3 months and mortality at 12 months.	[126]
at admission	6.4 ± 2.6 pg/mL (*n* = 11)	2.8 ± 1.5 pg/mL (*n* = 9) *p* = 0.002	Significant correlation of IL-6 with larger stroke volume (*p* = 0.012, r = 0.72,) and poorer prognosis at 1 year, as measured by ESS (*p* = 0.014, r = −0.71) and BI (*p* = 0.006, r = −0.80).	In acute ischemic stroke, IL-6 can be an early indicator of prognosis.	[92]
24–72 h	10.5 (6–27.25) pg/mL (*n* = 60)	9 (2.90–18) pg/mL (*n* = 123) *p* = 0.002	No differences in IL-6 levels at 24–72 h after stroke onset in non-lacunar versus lacunar strokes.	Higher IL-6 values were demonstrated compared to healthy individuals.	[94]
<20 h	9.7 (7.9–15.7) pg/mL (*n* = 50)	–	The level IL-6 was significantly higher in patients with better outcome (median 12.6 (7.6–21.4)) when compared with those with worse outcome (median 8.492.4–13.70 *p* < 0.0001). There was a significant inverse correlation between IL-6 and TNF-α levels in the entire cohort of patients (r = 0.78, *p* = 0.002)	Serum IL-6 concentration was inversely correlated with final neurological impairment and infarct size (*p* < 0.001). In patients with ischemic brain injury, IL-6 is associated with neuroprotection but not neurotoxicity.	[143]
50 h	13.78 ± 19.46 pg/mL (*n* = 60)	4.38 ± 15.88 pg/mL (*n* = 30) *p* = 0.002	The levels of serum IL-6 were significantly higher in stroke patients. IL-6 showed negative correlation with protein S (r = –0.504, *p* = 0.000) and CNS scores (r = –0.451, *p* = 0.000).	IL-6 was correlated with the worse clinical outcome.	[105]
at admission	8 (4–21) pg/mL (*n* = 68)	2 (2–4) pg/mL (*n* = 71) *p* = 0.035	IL-6 was higher in stroke patients than in controls. Higher IL-6 concentration was significantly associated with greater neurological deficit (NIHSS ≤ 16 (*n* = 30), 7 (4–11) pg/mL, NIHSS 17–22 (*n* = 24), 7 (5–16) pg/mL; NIHSS ≥ 23 (*n* = 17), 22 (10–71) pg/mL; *p* = 0.007) at admission and greater degree of patient disability assessed with BI (BI ≤ 29 (*n* = 28), 13 (6–28) pg/mL; BI 30–59 (*n* = 15), 8 (5–20) pg/mL; BI ≥ 60 (*n* = 28), 6 (4–12) pg/mL; *p* = 0.030) and mRS (mRS = 0–2 (*n* = 28), 6 (4–12) pg/mL; mRS = 3–4 (*n* = 14), 8 (5–11) pg/mL; mRS = 5–6 (*n* = 29), 13 (6–36) pg/mL; *p* = 0.019).	IL-6 levels measured 12 h after the onset of cerebral ischemia were higher in stroke patients compared to the control group, and this increase was associated with more severe stroke and worse stroke outcomes.	[104]
within <36 h of symptom onset	5.14 ± 4.98 pg/mL (mean ± SD) [3.18 (1.97–6.22) median (25–75%)] (*n* = 113)	–	The proportion of patients with severe stroke (NIHSS > 10) at admission rose in a graded fashion from 0% in patients with IL-6 in the bottom tertile (≤2.26 pg/mL) to 46% in those in the top tertile (>4.63 pg/mL). Similar trends were observed at day 1–2, day 4–5, and at discharge (*p* < 0.01). Disability during follow-up (mRS ≥ 2) rose from 13% for IL-6 in the bottom tertile to 48% in the top tertile (*p* = 0.01). Association with poor physical function (Stroke Impact Scale (SIS ≤ 85)) revealed a comparable trend rising from 32% at the bottom tertile to 63% at the top tertile (*p* = 0.04).	High plasma IL-6 levels are associated with stroke severity and poorer functional outcomes.	[129]
at admission	10 (6–28) pg/mL (*n* = 107)	8 (3.1–12) pg/mL (*n* = 102) *p* < 0.001	Significant association between IL-6 demographic variables (age), diagnostic subtype (lacunar (5 (2–8) pg/mL) or cardioembolic (12 (6.5–18) pg/mL; *p* = 0.003), and SSS score (b1= −0.068; *p* < 0.0001).	Significant association between IL-6 and severity of neurological deficit at admission, diagnostic subtype.	[93]
24 h	26.5 +/−2.3 pg/mL (range 6.4–161.3) (*n* = 46)	3.9 +/−1.5 pg/mL (range 2.3–5.9) (*n* = 98; *p* < 0.0001	The concentration of IL-6 in the blood of patients with acute ischemic stroke was significantly higher than in healthy controls.	There is an association between inflammation and circulating IL-6 levels in patients with acute hypoxic–ischemic injury, therefore IL-6 might be used as a warning signal, the concentration of which increases at an early inflammatory state.	[98]
24 h	<6.47 pg/mL (*n* = 200) lower NIHSS ≥6.47 pg/mL (*n* = 50) higher NIHSS	–	Plasma IL-6 concentration correlated with a more severe stroke as demonstrated by NIHSS and mRS (r = 0.318, *p* < 0.001 and r = 0.302, *p* < 0.001, respectively). IL-6 concentration was found to be the optimal predictor, with a cut point of 6.47, χ^2^ (I, *n*= 250) = 20.5, *p* < 0·001. The value of IL-6 above 6.47 during the acute phase predicted a subsequent lack of survival (*p*= 0.006, odds ratio 8.0).	IL-6 shows potential as an early signal of survival after acute ischemic stroke and suggests a clear cut-off point for high-risk patients who might benefit from closer clinical surveillance and/or therapeutic interventions.	[127]
2–6 h, 6 h, 24 h, 3 days, 7 days	Figure 1a in [99] ng/L (*n* = 69)	–	IL-6 showed different time courses depending on stroke outcome. The IL-6 levels were significantly increased in the patients with severe strokes compared to the mild/moderate stroke group as early as 6 h after stroke onset. The levels stayed elevated only until 24 h after symptom onset (6 h *p* = 0.022, 12 h *p* = 0.011, 24 h *p* = 0.006). IL-6 at 12 h, 24 h, and 3 days was independently related to the NIHSS. In the first days after stroke, IL-6 significantly correlated with CRP and MMP-9 at different time points, while only at 6 h after stroke onset, IL-6 correlated with MCP-1.	The data indicate significant differences in the early time course of several potential markers for the complex network of inflammation and brain damage after ischemic stroke, depending on the outcome.	[99]
72 h 6 months	No infection 1.4 ± 0.3 ng/mL (*n* = 37) Infection 1.6 ± 0.3 ng/mL (*n* = 45)	–	Plasma IL-6 at baseline was associated with decreased survival at 2 years (OR = 9.2, [95% CI 1.0, 85.1], *p* = 0.031) and this association was independent of SAI, severity of stroke, age and sex.	IL-6 is associated with SAI, after adjusting for recognized risk factors, and IL-6 is associated with mortality in the first two years post stroke.	[138]
at admission	4.6 (0.9 to 13.0) pg/mL (*n* = 175)	–	Strong association between IL-6 and poor stroke outcomes (favorable outcome (*n* = 111), 2.2 (0.6 to 5.5) *versus* poor outcome (*n* = 64), 12.1 (6.7 to 48.0); *p* < 0.001). The logistic regression analysis of individual blood markers after adjusting for age and initial NIHSS score revealed that plasma logIL-6 (adjusted OR: 1.75, 95% CI: 1.25 to 2.25, *p* = 0.001) reached a statistical significance. The statistical significance of logIL-6 remained after further adjustment for 72 h infarct volume and Afib (logIL-6, OR: 1.74, 95% CI: 1.24 to 2.44, *p* = 0.003), time of stroke onset (logIL-6, OR: 1.74, 95% CI: 1.24 to 2.44, *p* = 0.001) and previous statin use (logIL-6, OR: 1.81, 95% CI: 1.27 to 2.57, *p* = 0.001).	Circulating IL-6 levels have clinical value in predicting stroke outcomes.	[96]
1 day	42.92 ± 72.2 pg/mL	–	Statistically significant correlation between IL-6 and NIHSS and mRS of patients from the moment of admission to the end of the observation period (*p* < 0.001, r = 0.6). Statistically significant correlation between IL-6 and infarct size in brain magnetic resonance imaging (MRI) (*p* < 0.001, r = 0.7).	IL-6 contributes to determination of severity of ischemic stroke.	[91]
5 days	56.91 ± 82.63 pg/mL				
The samples were drawn on the day of randomization, but before the initiation of antiplatelet therapy	Age: <50 1.8 pg/mL (1.4–3.4) Age: 50–65 2.4 pg/mL (1.6–3.7) Age:>65–75 2.2 pg/mL (1.6–3.6) Age:>75 2.5 pg/mL (1.7–3.5) (*n* = 1244)	–	In unadjusted analyses, IL-6 was significantly related to the risk of having a recurrent ischemic stroke (adjusted HR per SD, 1.1; 95% confidence interval [CI], 1.0–1.2; *p* < 0.01. IL-6 was also associated with an increase in risk of major vascular events (adjusted HR per SD, 1.1; 95% CI, 1.02–1.19; *p* = 0.008). In categorical analyses, there was evidence that the treatment effect of dual antiplatelet therapy depended on IL-6 levels (*p* for interaction = 0.04 for major vascular events).	Among recent lacunar stroke patients, IL-6 concentration predict risk of recurrent vascular events, and they are associated with the effect of antiplatelet therapies.	[118]
48 h	15.82 ± 16.64 pg/mL (*n* = 42)	6.64 ± 2.5 (*n* = 34) *p* = 0.000035	Patients with early ischemic stroke had significantly higher serum IL-6 levels than controls.	Ischemic stroke is associated with higher serum IL-6 levels.	[102]
at admission	21.91 ±30.75 pg/mL (*n* = 33)	1.04 ± 1.12 pg/mL (*n* = 60)	Baseline levels of IL-6 and stroke severity: The higher baseline IL-6 level showed a significant positive correlation with the NIHSS score at day 1(r^2^ = 0.18; *p* = 0.01), on day 7 following admission (r^2^ = 0.20; *p* = 0.01) and with the infarct volume on DWI (r^2^ = 0.17; *p* = 0.01). IL-6 was found to correlate with worse long-term outcomes at both 1 month (r^2^ = 0.19; *p* = 0.01) and 3 months (r^2^ = 0.15; *p* = 0.04), as measured by MRS. The baseline IL-6 levels was independent predictors of short term outcome as assessed by NIHSS at day 7 (*p* = 0.04); also for long term outcome as measured by MRS at one month (*p* = 0.02) and three months *(p* = 0.04). Baseline levels of IL-6 were higher (*p* = 0.04) in those patients who died than those who survived.	Serum IL-6 concentrations were significantly higher in patients with ischemic stroke than in the healthy control group. A correlation was observed between the clinical and radiological severity of stroke and the level of IL-6, and the poor outcome could be predicted (the higher baseline IL-6 may predict mortality in ischemic stroke). This suggests that the IL-6 serum may be a potentially useful inflammatory biomarker for the prognosis of acute ischemic stroke.	[95]
27 days	12.80 ±21.18 pg/mL (*n* = 33)				
1 month	6.18 ±14.21 pg/mL (*n* = 33)				
3 months	2.75 ± 2.54 pg/mL (*n* = 33)				
24 h	2.61 (1.60–4.73) pg/mL (*n* = 7053)	–	Stroke recurrence was observed in 458 (6.5%) patients, and functional disability was seen in 1708 (24.2%) patients at the 90-day follow-up. Per stand deviation (4.26 pg/mL) increase in IL-6 concentration was associated with a significantly 19% increased risk of stroke recurrence (OR, 1.19; 95% CI, 1.09–1.29) and a significantly 22% increased risk of disability (OR, 1.22; 95% CI, 1.15–1.30) at 90 days. The mediated proportion of the association between IL-6 and functional disability by stroke recurrence was 18.72% (95% CI, 9.26–28.18%) in the adjusted model.	Stroke recurrence mediates less than 20% of the association between IL-6 and functional outcome at 90 days among patients with acute ischemic stroke.	[114]
–	Lacunar stroke 6.6 (7.5) pg/mL (*n* = 52)	–	Baseline IL-6 was significantly associated with risk of main outcome in them ultivariable analysis, after adjustment for age, sex, and small vessel disease score (1.4 (1.02–2.2); *p* = 0.04). IL-6 was significantly related to along-term risk of vascular events or death in patients with cerebral small vessel disease (CSVD).	It demonstrated the important prognostic role of Il-6 in persons with different clinical manifestations of CSVD. The strongest association occurred between IL-6 and recurrent stroke, other vascular events and death.	[115]
–	4.5 (3.1) ng/mL (*n* = 557)	cohort random sample 3.7 (2.6) ng/mL (*n* = 951) *p* < 0.001	IL-6 was associated with risk factors for stroke. After adjusting for different factors (age, sex, race) the hazard ratio (HR; 95% CI) for incident stroke for the highest versus lowest quartile of IL-6 was 2.4 (1.6–3.4). IL-6 mediated the black-white disparity in stroke risk, but mediation was mediated by associations of IL-6 with stroke risk factors.	IL-6 was strongly associated with stroke risk factors and a substantially increased risk of incident stroke, independent of racial disparity.	[16]
at admission	Intra-hospital improvement ^h^/extra-hospital improvement ^i^	–	Th high levels of IL-6 on admission had a negative correlation with extrahospital improvement (β: −0.305, *p* < 0.0001), while high levels of IL6 at 24 hours correlates with a negative intrahospital improvement and a positive extrahospital improvement (β:−0.121, *p* < 0.0001 vs. β: 0.131, *p* < 0.0001).	High IL-6 level at 24 hours was associated with a worse intra-hospital improvement and with better extra-hospital improvement. The variations in IL6 level in the first 24 hours clearly showed a relationship between the molecular components of the ischemic cascade and the clinical outcome of patients.	[136]
	21.1 ± 15.8/22.4 ± 15.3 (NO *n* = 1176/1326) 21.3 ± 15.2/20.1 ± 15.3 (YES *n* = 3119/2527) *p* = 0.844/0.043				
24 h	62.2 ± 47.5/20.1 ± 25.0 (NO *n* = 1176/1326) 51.1 ± 46.3/96.5 ± 30.8 (YES *n* = 3119/2527) *p* = 0.006/<0.0001				
24 h	18.8 (4.0) pg/mL (*n* = 176)	6.7 (4.6) pg/mL (*n* = 176) *p* < 0.001	The results showed that the best predictors of IS were SBP, glucose, NOx, hydroperoxides, IL-6, and WBC (all positively associated), 25(OH)D (negatively associated), and sex (male) (x^2^ = 159.64, df = 8, *p* < 0.001, Nagelkerke = 0.787) and that 89.4% of all cases were correctly classified with sensitivity of 86.2% and specificity of 93.0%. IL-6 is significantly higher in patients with an endpoint mRS ≥ 3 (27.5 (4.1) pg/mL) vs. those with an endpoint mRS < 3 (8.7(7.2) pg/mL; *p* < 0.001).	The cumulative effects of immune-inflammatory, metabolic, oxidative, and nitrosative stress (IMO&NS) biomarkers are associated with AIS and predict a poor outcome at 3-month follow-up.	[107]
<7 days	Figure 2a,b in [106]	–	Baseline IL-6 was associated with poor functional outcome (odds ratio [OR] per 1-unit increase 1.02, CI 1.01–1.04, *p* = 0.002). After adjustment for age, sex, qualifying event, baseline mRS, hypertension, anti-platelet and statins on discharge, the association remained unchanged (adjusted OR 1.02, CI 1.01–1.04, *p* = 0.004). A relationship between IL-6 and mortality after 1 year was observed. When the highest quartile was compared to the lowest quartile IL-6, the HR was 2.30 (CI 1.10–4.84, *p* = 0.03)	Baseline inflammatory cytokines independently predicted late recurrence, supporting a rationale for randomized trials of anti-inflammatory agents for prevention after stroke and suggesting that targeted therapy to high-risk patients with high baseline inflammation may be beneficial.	[106]
at admission	^a^ 1.3 [0.3–3.6] pg/mL	–	High IL-6 levels at 6, 24, and 48 h, in combination with a higher age, a pre-stroke mRS score > 2, a past history of hypertension or diabetes, current smoking, a higher baseline National Institute of Health Stroke Scale Score, the absence of associated intravenous thrombolysis, an intracranial internal carotid artery or a tandem occlusion and an increased infarct growth were associated with futile reperfusion.	IL-6 is a marker of futile reperfusion in the setting of MT.	[121]
	^b^ 1.3 [0.7–3.4] pg/mL				
	^c^ 2.0 [0.7–4.5] pg/mL				
6 h	^a^ 3.1 [2.0–6.0] pg/mL				
	^b^ 2.1 [1.1–4.4] pg/mL				
	^c^ 3.3 [2.2–6.2] pg/mL				
24 h	^a^ 5.0 [3.3–7.3] pg/mL				
	^b^ 2.7 [1.7–5.5] pg/mL				
	^c^ 4.3 [2.6–9.0] pg/mL				
48 h	^a^ 5.2 [2.9–15.9] pg/mL				
	^b^ 2.5 [1.2–5.2] pg/mL				
	^c^ 5.3 [2.5–8.2] pg/mL				
3 months	^a^ 1.2 [0.3–2.0] pg/mL				
	^b^ 0.6 [0.3–1.1] pg/mL				
	^c^ 1.0 [0.4–2.6] pg/mL				
24 h	(4.3; 8; 14.9) pg/mL (*n* = 332)	–	In linear regression analysis that was adjusted for etiology, IL-6 was found to be a biomarker independently associated with infarct volume (Atheromatous, (*n* = 59), 8.9 (4.9–14.6); Cardioembolic (*n* = 126), 10.9 (6.1–23.4); Small vessel (*n* = 63), 4.8 (2.8–9.7); Indeterminate, (*n* = 77), 8.1 (5.4–14.7); Unusual, (*n* = 7), 4.5 (1.9–10.0); *p* < 0.001).	IL-6 levels may be independently associated with infarct volume. The results may have clinical significance because IL-6 levels in blood may indicate the extension of brain infarction without the need to use neuroimaging techniques.	[124]
27–96 h	IL-6: Q1: ≤1.6 ng/L (*n* = 2896) Q2: 1.6–2.6 ng/L (*n* = 1872) Q3: 2.6–5.1 ng/mL (*n* = 1934) Q4: >5.1 ng/mL (*n* = 3051)	–	The highest quartiles of IL-6 (adjusted HR, 1.36; 95% CI 1.13–1.64; *p* = 0.001) were associated with increased risk of recurrent stroke; and the highest quartiles of IL-6 (adjusted OR 1.93; 95% CI 1.64–2.27; *p* < 0.0001), were correlated with increased risk of poor functional outcome.	In patients with ischemic stroke, an association was found between IL-6 and recurrent stroke, composite vascular events, and poor functional outcome.	[45]
Oset, 24 h, 72 h	poor outcome (mRS ≥ 2)/ good outcome after 90 days 8.72 (IQR 3.04–26.89) pg/mL/ 3.72 (IQR, 1.88–8.58) pg/mL *p* = 0.012	–	The level of IL-6 was significantly increased in the NIHSS > 5 group at admission (*p* < 0.001) compared to the NIHSS ≤ 5 group. IL-6 was notably higher in the poor outcome group (*p* = 0.012). IL-6 was an independent predictor of risk for AIS patients with an adjusted OR of 1.152 (*p* = 0.028). Patients with a NIHSS score of less than 5 exhibited lower IL-6 levels, indicating that increased levels of IL-6 correlated with AIS severity after intravenous thrombolytic treatment (IVT).	IL-6 might be useful plasma marker to predict the prognosis in AIS patients at 90 days after IVT.	[116]
4.5 h	6.37 (4.35; 7.43) pg/mL (*n* = 125)	3.59(3.42; 4.04) (*n* = 28; *p* < 0.001)	Correlations were found between IL-6 serum concentration measured during the onset and on the 1st day of ischemic stroke and the NIHSS results assessed on admission (R = 0.43, *p* < 0.01 and R = 0.4, *p* < 0.01, respectively) and at discharge (R = 0.61, *p* < 0.01 and R = 0.52, *p* < 0.01, respectively. Moreover, an association was observed between IL-6 serum levels determined in <4.5 h and on the 1st day of ischemic stroke, and results evaluated according to the mRS scale on admission (R = 0.52, *p* < 0.01 and R = 0.44, *p* < 0.01, respectively), at discharge (R = 0.61, *p* < 0.01 and R = 0.47, *p* < 0.01, respectively), after 3 months (R = 0.68, *p* < 0.01 and R = 0.3, *p* < 0.01, respectively), and 1 year since the stroke (R = 0.73, *p* < 0.01 and R = 0.50, *p* < 0.01, respectively) and IL-6 concentration measured 7 days after discharge (R = 0.28, *p* = 0.04). The significant statistical connection between IL-6 levels during onset and TNF-α levels assessed in <4.5 h and after 24 h since the stroke (*p* < 0.01) were detected. The IL-6 (5.59 pg/mL vs. 7.29 pg/mL) with *p* = 0.016 assessed in time < 4.5 h was statistically lower in patients suffering from the LACI (lacunar cerebral infarct) ischemic stroke in comparison to the patients with PACI (partial anterior circulation infarct) stroke. In the case of IL-6 level measured on the 7th day (>4 pg/mL), an increase in mortality was observed.	IL-6 levels were higher compared to the control group and in the subgroup of patients with an unfavorable functional outcome (mRS: 3–6 points). A positive correlation was found between IL-6 and TNF-α levels in patients with AIS within <4.5 h and on the day of stroke. Higher IL-6 levels in the acute phase of stroke and on the first and seventh day were associated with a worse early and late prognosis in patients treated with intravenous thrombolysis. An association between IL-6 levels in subacute AIS and the severity of neurological deficit was shown. IL-6 may be a prognostic factor in thrombolytic treatment of AIS.	[2]
24 h	7.09 (6.48; 9.30) pg/L (*n* = 125)				
7 days	6.54 (4.44; 7.58) pg/mL (*n* = 125)				
at admission	FPE <3.0 pg/mL (*n* = 43) Figure 2 in [122]	Non-FPE (*n* = 108) *p* < 0.05 Figure 2 in [122].	FPE was found to be associated with low IL-6 levels and platelet count, an older age, a lack of hypertension, a lack of tandem occlusion, a shorter thrombus length, and a reduced procedural time. Multivariate analysis showed that low IL-6 admission level was associated with FPE (OR 0.66, 95% CI 0.46 to 0.94). Optimal cut-off of IL-6 level for distinguishing FPE from non-FPE was 3.0 pg/mL (sensitivity 92.3%, specificity 42.3%).	A lower admission level of IL-6 is associated with FPE.	[122]
-	310.7 ± 110.6 pg/mL (155–522) (*n* = 45)	34.2 ± 14.3 pg/mL (13–62) (*n* = 45) *p* < 0.0001	The patient subgroups had significantly different levels of IL-6 as the severe cases had the highest level and mild ones had the lowest level (*p* < 0.001). There was a significant negative correlation between serum miR 221 with NIHSS score and IL-6 while there was a significant positive correlation with the grade of weakness (r = −0.75, *p* < 0.0001, r = −0.74, *p* < 0.0001 and r = 0.79, *p* < 0.001, respectively). The ROC curve with AUC of 0.99 for serum miR-221 combined with IL-6, showed both higher sensitivity and specificity of 96.4% and 89%, respectively.	Serum level of miR-221 may be considered a sensitive and specific marker for diagnosis and for assessing the severity of ischemic stroke especially if combined with IL-6. Such markers could be recommended as diagnostic tools and early predictive markers for the severity of the ischemic stroke disease.	[123]
<24 h	Table 1 in [137].	–	IL-6 were significantly correlated (*p* < 0.05) with disability assessed by mRS score. Higher intracranial levels of IL-6 were associated with a lower likelihood (mRS score at 90 days > 2) of favorable outcome (adjusted OR per SD increase, 0.51; 95% CI 0.27 to 0.95).	The correlation between IL-6 and mRS score outcome support an avenue for add-on and localized immune modulatory strategies in AIS.	[137]
-	LAA 3.7 (2.2–8.2) pg/mL; (*n* = 272) ^d^/3.1(1.9–5.7); (*n* = 1467) ^e^ CE 6.0(3.0–12.1) pg/mL; (*n* = 85) ^d^/4.3 (2.1–7.7); (*n* = 369) ^e^ SAO 2.5 (1.6–4.9) pg/mL; (*n* = 173) ^d^/2.2 (1.4–3.7); (*n* = 1397) ^e^	–	At 3 months poststroke, 1026 (14.8%) patients experienced worse outcomes. Compared with patients with good outcomes, patients with poorer outcomes had significantly higher levels of inflammatory biomarkers, including IL-6 (*p* < 0.05). The highest quartile of IL-6 (1.43 [1.16–1.76]) was associated with an increased risk of worse outcomes at 3 months.	Significant associations have been found between elevated levels of inflammatory markers, including IL-6, and increased risk of poorer outcomes in patients with acute ischemic stroke. Targeting the inflammatory response, through novel anti-inflammatory therapies, may hold promise for improving neurological outcomes in stroke patients.	[113]
24 h	Mean ± S 3.8 ± 4.0 pg/mL Median (IQR) 2.4(1.5–4.2) pg/mL; (*n* = 3910) ^f^ Mean ± S 5.7 ± 5.7 pg/mL Median (IQR) 2.8(1.9–8.1) pg/mL; (*n* = 121) ^g^	–	Patients with ND had higher levels of IL-6 (median, 2.4 vs. 2.8 pg/mL) than patients without ND. IL-6 levels (adjusted OR, 1.43 [95% CI, 1.24–1.66]) remained independent predictor of in-hospital ND. The above relationship was also observed in long-term poor outcomes, including unfavorable functional outcomes (defined as mRS 2–5) and death.	Elevated IL-6 was an independent predictor for ND in minor AIS patients with LAA and SVO subtypes. IL-6 had the most notable incremental predictive value of in-hospital ND in addition to conventional predictors, both collectively and in the LAA subgroup.	[103]

^a^ No or partial reperfusion (*n* = 31); ^b^ successful reperfusion—effective (*n* = 84); ^c^ successful reperfusion—futile (*n* = 46);^d^ worse outcome (worse outcomes at 3 months were defined as ΔmRS_3m-discharge_ ≥ 1 (ΔmRS_3m-discharge_ = mRS_3m-mRSdischarge_); ^e^ good outcome; ^f^ no ND, neurological deterioration; ^g^ ND, neurological deterioration; ^h^ positive value of the NIHSS on admission minus the value of the NIHSS at discharge. ^i^ positive value of the NIHSS scale at discharge minus the value of the NIHSS scale at three months.

## Data Availability

Not applicable.
